# Dengue and SARS-CoV-2 co-circulation and overlapping infections in hospitalized patients

**DOI:** 10.3389/fcimb.2024.1429309

**Published:** 2024-11-08

**Authors:** Thayza M. I. L. Santos, Alice F. Versiani, Guilherme R. F. Campos, Marilia M. Moraes, Maisa C. P. Parra, Natalia F. B. Mistrao, Andreia F. Negri, Flavia F. Bagno, Marina G. Galves, Camila M. Moreno, Flavio G. Da Fonseca, Cassia F. Estofolete, Nikos Vasilakis, Mauricio L. Nogueira

**Affiliations:** ^1^ Laboratório de Pesquisa em Virologia, Departamento de Doenças Infecciosas e Parasitárias, Faculdade de Medicina de São José do Rio Preto, São José do Rio Preto, Brazil; ^2^ Department of Pathology, University of Texas Medical Branch, Galveston, TX, United States; ^3^ Prefeitura de São José do Rio Preto, Vigilância Epidemiológica, São José do Rio Preto, Brazil; ^4^ Centro de Tecnologia em Vacinas, Universidade Federal de Minas Gerais, Belo Horizonte, Brazil; ^5^ Laboratorio de Virologia Basica e Aplicada, Departamento de Microbiologia, Universidade Federal de Minas Gerais, Belo Horizonte, Brazil; ^6^ Center for Vector-Borne and Zoonotic Diseases, University of Texas Medical Branch, Galveston, TX, United States; ^7^ Institute for Human Infections and Immunity, University of Texas Medical Branch, Galveston, TX, United States

**Keywords:** dengue virus, SARS-CoV-2, overlapping infection, arboviruses, epidemiology

## Abstract

Since its emergence in 2019, coronavirus disease (COVID-19) has spread worldwide and consumed public health resources. However, the world still has to address the burdens of other infectious diseases that continue to thrive. Countries in the tropics and neotropics, including Brazil, are affected by annual, cyclic dengue epidemics. Little is known about the impact of subsequent infections between DENV and SARS-CoV-2. Our study was performed on 400 serum samples collected from laboratory-confirmed COVID-19 patients between January and June 2021, months historically known for DENV outbreaks in Brazil. The samples were tested by serology and molecular assays for the presence of DENV and other arboviruses. While no DENV PCR results were detected, 6% were DENV IgM-positive, and 0.25% were DENV NS1-positive according to ELISA. IgM antibodies were isolated by chromatography, and 62.5% of the samples were positive for neutralizing antibodies (FRNT_80_) against DENV IgM, suggesting a recent infection. We also observed increased IL-10, TNF-α, and IL-1β levels in patients with overlapping SARS-CoV-2/DENV infections. Intriguingly, diabetes was the only relevant comorbidity (p=0.046). High rates of hospitalization (94.9%) and mortality (50%) were found, with a significant increase in invasive mechanical ventilatory support (86.96%) in SARS-CoV-2/DENV- infected patients, suggesting an impact on patient clinical outcomes. When analyzing previous exposure to DENV, secondary dengue patients infected with SARS-CoV-2 more frequently presented with dyspnea and respiratory distress, longer hospital and intensive care unit (ICU) stays (4 and 20.29 days, respectively) and a higher mortality rate (60%). However, a greater proportion of patients with primary DENV infection had fever and cough than patients with secondary dengue (87.50% vs. 33.33%, p=0.027 for fever). Our data demonstrate that differentiating between the two diseases is a great concern for tropical countries and should be explored to improve patient management.

## 1 Introduction

Since the emergence of the novel severe acute respiratory syndrome coronavirus 2 (SARS-CoV-2) in late 2019, the coronavirus disease (COVID-19) it causes has spread worldwide, and the World Health Organization (WHO) declared it a pandemic event and a Public Health Emergency of International Concern (PHEIC) on 30 January 2020 (Eurosurveillance Editorial Team, 2020). To date, more than 767 million people have been infected, resulting in almost 7 million deaths worldwide (World Health Organization, 2020). Although first described as a respiratory disease, COVID-19 is now recognized as a multiorgan disease with a broad spectrum of manifestations. SARS-CoV-2 infection can remain asymptomatic, and symptomatic disease may present as a spectrum of symptoms ranging from mild flu-like symptoms to severe pneumonia, dyspnea, organ dysfunction, and death (Zhang et al., 2023). At least 10% of severe SARS-CoV-2 manifestations can lead to a series of debilitating chronic illnesses, now called long COVID-19 (or post-acute sequelae of COVID-19) (Davis et al., 2023). Despite the global impairment of health services due to COVID-19, the world still needs to address the burdens of various other diseases that present with comparable symptoms, leading to misdiagnoses, unsuitable treatments, and unpredictable consequences on outcome severity in coinfections (Nacher et al., 2020; Estofolete et al., 2021; Gil et al., 2021; Swets et al., 2022; Wang et al., 2022). In tropical and neotropical regions, such as Brazil, where arboviral diseases are hyperendemic, the chances of arbovirus coinfection have significantly increased due to the rapid and uncontrolled spread of SARS-CoV-2 (Lu et al., 2021; Schulte et al., 2021).

Among arboviral infections, dengue viruses (DENVs) are by far the most important mosquito-borne pathogens in the tropics around the world, putting nearly half of the global human population at risk of infection (Bhatt et al., 2013; Messina et al., 2019). DENVs are grouped into four antigenically distinct serotypes (DENV1-4), each comprising 4-7 genotypes with multiple lineages, and new lineages are often associated with new outbreaks (Chen and Vasilakis, 2011; Guzman and Harris, 2015). Most DENV infections are asymptomatic, whereas symptomatic infections often present as a self-limited flu-like illness characterized by a sudden onset of fever, arthralgia, myalgia, retro-orbital headaches, maculopapular rash, and leukopenia [commonly referred to as classic dengue fever (DF)]. Moreover, some DF patients progress to severe disease [severe dengue disease (SDD)], characterized by vascular leakage and/or hemorrhage, causing up to 500,000 annual hospitalizations with a fatality rate of approximately 3% (World Health Organization, 2009; St John and Rathore, 2019; Wilder-Smith et al., 2019). However, risk factors for progression to SDD may depend on prior exposure to a heterologous DENV infection, age, nutrition, viral genetics, and host genetic predisposition (Chen and Vasilakis, 2011; St John and Rathore, 2019).

São José do Rio Preto, a city located in the northwest of the state of São Paulo, is known as a hyperendemic area for DENV. It bears the occurrence of cyclical epidemics of different serotypes and a high rate of infestation by *Aedes aegypti*, the main vector of transmission for multiple arboviruses, including Zika and chikungunya (Colombo et al., 2016; Chiaravalloti-Neto et al., 2019; Estofolete et al., 2019), which are actively transmitted in the region (Estofolete et al., 2019). Several studies have demonstrated similarities in the clinical presentations and laboratory observations of DENV and COVID-19 infections (Silvestre et al., 2021). However, little is known about the impact of overlapping infections with these two viruses on patient clinical manifestations, the potential for severe disease progression, and patient prognosis (Estofolete et al., 2021). In our study, we investigated the occurrence of dengue and COVID-19 sequential infections by analyzing surveillance databases and an acute COVID-19 hospital cohort.

## 2 Materials and methods

### 2.1 Epidemiological data sources and ethics statement

We retrospectively analyzed a cohort of patients who were diagnosed with dengue and/or COVID-19 between January and July 2021 in Sao Jose do Rio Preto municipality. The data were retrieved from the Reporting Disease Information System (SINAN), which is maintained by the Brazilian Ministry of Health and includes all relevant information regarding notifiable diseases. For COVID-19 patients, we obtained information from databases of both mild respiratory syndrome (e-SUS) and severe acute respiratory syndrome (SRAG) patients. These databases are available online and have registered all patients in the Epidemiologic Surveillance System for all Brazilian states.

### 2.2 Moving average analysis

A time-trend analysis was performed using a seven-day moving average of confirmed cases of dengue, COVID-19, and the reported overlapping occurrences and coinfection cases identified in the SJdRP reporting system from January to July 2021. The data were retrieved from the Public Health System of SJdRP and received from the Reporting Disease Information System (SINAN).

### 2.3 Geoprocessing

All Dengue and SARS-CoV-2 case data were registered in specific databases in Microsoft Excel software. The databases included the addresses, dates on which the cases were reported, and assays that were performed. We used TerraView 4.2.2 (INPE, Sao Paulo, SP, BR) and ArcGIS 10.6.1 (ESRI, Redland, CA, USA) software for geocoding and spatial analysis. All the shapefile data were downloaded from https://portaldemapas.ibge.gov.br/portal.php#mapa218541. These downloads are available on a Brazilian government site and are free for the world community.

### 2.4 Hospital cohort

In this study, we also investigated the presence of overlapping infection with DENV in serum samples from patients with a positive diagnosis of COVID-19 who sought healthcare services at a local university hospital, Hospital de Base (HB). HB is a reference health center and is one of the hospital complexes piloting COVID-19 care and treatment in São Paulo state, and it has the second largest COVID-19 ICU in Brazil. The hospital is linked to the Faculdade de Medicina de São José do Rio Preto (FAMERP), an educational facility where Laboratório de Pesquisas em Virologia (LPV) is located and where this research was conducted.

From January to June 2021, 400 samples were collected from patients at the time of admission to the hospital and during treatment. Nasopharyngeal swabs were collected during admission and submitted to SARS-CoV-2 molecular investigation upon to arrival. Patients were admitted directly on the hospital or recommended from other clinical facilities due to disease progression. Sera were collected during hospitalization, and/or ICU admission. Only patients with paired samples were selected for this study. The selection of patients was random on the purpose to mimic population co-circulation dynamics. All samples were kept on -80°C ultra freezer up to following analyses. Samples post antibody depletion by protein G column were maintained on -20°C freezer prior neutralization assay. Negative samples were obtained from the hospital sera bank from patients without dengue-like or COVID-19-like symptoms within the study time period. The data collected from hospital medical records were compared with municipal epidemiological surveillance data. The database included demographic information, main signs and symptoms at admission, days of symptoms, comorbidities, patient hospitalization exams, disease progression records, mechanical ventilatory support, and COVID-19 vaccination status.

### 2.5 Respiratory samples and molecular investigation

Nasopharyngeal swab samples from patients admitted to HB are included in routine COVID-19 diagnosis. From these swabs, viral RNA was extracted from 140 µL of nasopharyngeal samples using a QIAmp Viral RNA Mini Kit (QIAGEN, Hilden, Germany) according to the manufacturer’s protocols. SARS-CoV-2 RNA was analyzed by one-step real-time polymerase chain reaction (RT−qPCR) using primers and probes targeting the envelope (E) or the nucleocapsid (N) region of the SARS-CoV-2 genome and human RNAse P according to the instructions of the GeneFinder COVID-19 Plus RealAmp Kit (OSANG Healthcare Co., Gyeonggi, South Korea) (OSANGHealthcare, 2021). Sera samples were tested by RT−qPCR Multiplex for the 4 DENV serotypes, as well as for Zika virus (ZIKV) and Chikungunya virus (CHIKV), to determine the presence of those viruses. Viral RNA was extracted using a Zymo Quick-RNA Viral Kit R1035 (Zymo Research, Irvine, CA, USA). Both a CDC DENV 1-4 RT−qPCR multiplex assay and a ZDC Molecular Kit (Instituto de Tecnologia em Imunobiológicos Biomanguinhos, BRA) were performed for the detection of ZIKV, DENV, and CHIKV according to the manufacturer’s specifications in a StepOnePlus™ Real-Time PCR System (Applied Biosystems^®^, USA) (Johnson et al., 2005; Ribeiro et al., 2021). RT−qPCR was also conducted in a QuantStudio 3 Real-Time PCR System (Thermo Fisher Scientific, USA). The results were analyzed in QuantStudio 3 software v1.5.1 (Thermo Fisher Scientific, USA) and were interpreted as having a cycle quantification value (Cq) less than or equal to 40 (positive) and a Cq greater than 40 (negative).

### 2.6 COVID-19 serological investigation

An ELISA for COVID-19 IgG (CT-Vacinas, Brazil) was performed for the qualitative determination of IgG levels against the Nucleocapsid (N) (Bagno et al., 2022) and Spike (S) proteins of SARS-CoV-2. For this, 5 µL of controls and 5 µL of each sample were added to 500 µL of sample diluent in previously identified individual microtubes or dilution plates. The solution was gently mixed, and 100 µL of each positive control, negative control, or diluted sample was added to each well of the plate. This mixture was then covered with a plate sealer and incubated for 30 min at 37 ± 2°C so that the SARS-CoV-2-specific antibodies present in the sample bound to the recombinant antigen immobilized on the microplate, forming the specific antigen-antibody complex. After the initial incubation, during which the protein diluted in the buffer was adsorbed to the plate, it was washed five times with 10X washing solution previously diluted 1:10 to remove unbound materials. After washing, 100 µL of peroxidase-conjugated anti-human IgG solution was added to each well of the microplate and incubated for 30 min at 37 ± 2°C, thus allowing the binding of this anti-IgG conjugated to the antigen-antibody complexes (protein + specific IgG). Another wash was performed to remove unbound complexes. After this step, 100 µL of substrate for the peroxidase enzyme was added, and the mixture was incubated, resulting in a blue color, which indicated the presence of anti-SARS-CoV-2 IgG antibodies in the samples. Finally, 100 µL of stop solution was added to interrupt the reaction, and a color change from blue to yellow occurred. The absorbance of the reaction was measured in a microplate reader at 450 nm. The reference values for the controls were as follows: Blank (Br) < 0.60, Negative Control (C-) 0.60 ≤ C- ≤ 0.250, and Positive Control (C+) 0.7 ≤ C+ ≤ 0.20. The cutoff was considered the value obtained by the formula (C**0.1)+0.18, where C* is the mean of the absorbances obtained with the positive control. The index was calculated by dividing the sample absorbance by the cutoff value. The final result was interpreted as nonreactive < 0.80 for the qualitative index, reactive > 1.10, or indeterminate 0.80-1.09. All ELISAs were performed on a SpectraMax Plus Microplate Reader (Molecular Devices, USA). Graphs and statistical analyses were performed using GraphPad Prism, version 9.0 (GraphPad Software, San Diego, CA, USA).

### 2.7 DENV serological investigation

A Panbio™ Dengue Early Elisa (NS1), Panbio™ Dengue IgM Capture ELISA, and a Panbio™ Dengue IgG Indirect ELISA (Abbott Laboratories, Abbott Park, IL, USA) were performed following the manufacturer’s specifications and read on a SpectraMax Plus Microplate Reader (Molecular Devices, USA) to determine the presence of the NS1 antigen and DENV IgM and IgG antibodies in the serum samples, respectively. Graphs and statistical analyses were performed using GraphPad Prism, version 9.0 (GraphPad Software, San Diego, CA, USA).

### 2.8 Antibody depletion by a protein G column and purification

DENV IgM and IgG antibodies from serum-positive patients were isolated by using Pierce™ Chromatography Cartridges using a Protein G column according to the manufacturer’s specifications (**Supplementary Figure 1**). A selection of samples of these isolated positive DENV M and G antibodies were concentrated by ultrafiltration using a Vivaspin^®^ 2 (Sartorius, Göttingen, Germany). The protocol used followed the manufacturer’s specifications. Antibody purification was confirmed by a Panbio™ DENGUE IgM Capture ELISA and a Panbio™ DENGUE IgG INDIRECT ELISA (Abbott Laboratories, Abbott Park, IL, USA), both of which conform to the manufacturer’s recommendations.

### 2.9 Focus reduction neutralization test (FRNT50 and 80)

DENV1-4 FRNTs were grown in 24-well Costar^®^ (Corning, USA) plates using a fixed virus inoculum [∼100 focus forming units (FFU) per well] against a fixed serum dilution (1:20). Serum samples were diluted in Dulbecco’s modified Eagle’s medium (DMEM) supplemented with 2% FBS and antibiotics (Gibco, USA). The virus was mixed with an equal volume of each serum dilution, and the mixture was incubated for 1 h at 37°C/5% CO_2_. Then, 100 μL of the serum-virus mixture was added to Vero cultures and incubated for 1 h at 37°C/5% CO_2_. The wells were then overlaid with 1 ml of 0.8% methylcellulose (Sigma−Aldrich, St. Louis) diluted in warm DMEM (Gibco, USA) supplemented with 2% FBS and antibiotics and incubated undisturbed for 4 days at 37°C/5% CO_2_. The methylcellulose overlay was aspirated, followed by fixation of the cell monolayer with a mixture of ice-cold acetone and methanol (1:1) solution and incubation for 1 h at room temperature (RT). The fixation solution was aspirated, and the plates were washed and allowed to air dry. The plates were washed three times with 1× PBS and blocked with PBS supplemented with 3% FBS for 15 min at RT, followed by overnight incubation with anti-DENV-2 antibody. The plates were washed three times, followed by an hour-long incubation with a secondary antibody conjugated to horseradish peroxidase (KPL, Gaithersburg, MD). Detection proceeded with the addition of aminoethylcarbazole (AEC) substrate (ENZO Life Sciences, Farmingdale, CT) prepared according to the manufacturer’s instructions. The PRNT titers were scored as the reciprocal of the serum dilution able to inhibit 50 and 80% of foci.

### 2.10 Evaluation of cytokine expression profiles

A commercial panel was used to determine the cytokine levels in the patient serum samples. A subgroup of SARS-CoV-2- and DENV-positive samples with severe disease manifestations (ICU patients) confirmed by FRNT (n=8) was compared with samples positive for only COVID-19 (n = 16), and a cytokine assay was performed using Invitrogen™ Human HS ProcartaPlex^®^ PI-5 (Thermo Fisher Scientific, USA) for the detection and measurement of IL-1β, IL-6, IL-8, IL-10, and TNF-α levels. The samples were analyzed by the xPONENT MAGPIX program on MAGPIX^®^ from LUMINEX^®^ according to the manufacturer’s instructions. Negative samples (n=16) were obtained from the hospital sera bank and validated as described for the positive groups (molecular e serology). The standard curves of known concentrations of recombinant human cytokines were used to convert fluorescence units into cytokine concentration units (pg/mL). The data were stored and analyzed using GraphPad Prism software, version 9.0 (GraphPad Software, San Diego, CA, USA). Statistical analysis was performed with ordinary one-way ANOVA with Bonferroni correction as a multiple-hypothesis test.

### 2.11 Statistical analysis

After confirmation of infection by DENV and/or SARS-CoV-2, the demographic and clinical manifestations of the patients were evaluated, and statistically significant differences were considered at p <0.05. All analyses were performed using IBM SPSS software for iOS (version 28, SPSS, Chicago, IL, USA). Pearson’s chi-square or Fisher’s exact tests were used to determine whether the expected frequency in the groups was met, and then the associations between categorical variables were established. For continuous variables, the normality of the distribution was tested using the Shapiro−Wilk test. ANOVA with the Bonferroni *post hoc* correction or t-test was used to evaluate the significance of differences between each group in normally distributed data. When the data were not normally distributed, the Kruskal−Wallis or Mann−Whitney test was used to determine whether there was a difference between the means of the groups.

## 3 Results

### 3.1 Epidemiologic profile of overlapping infection with DENV and SARS-CoV-2

**Figure 1:**
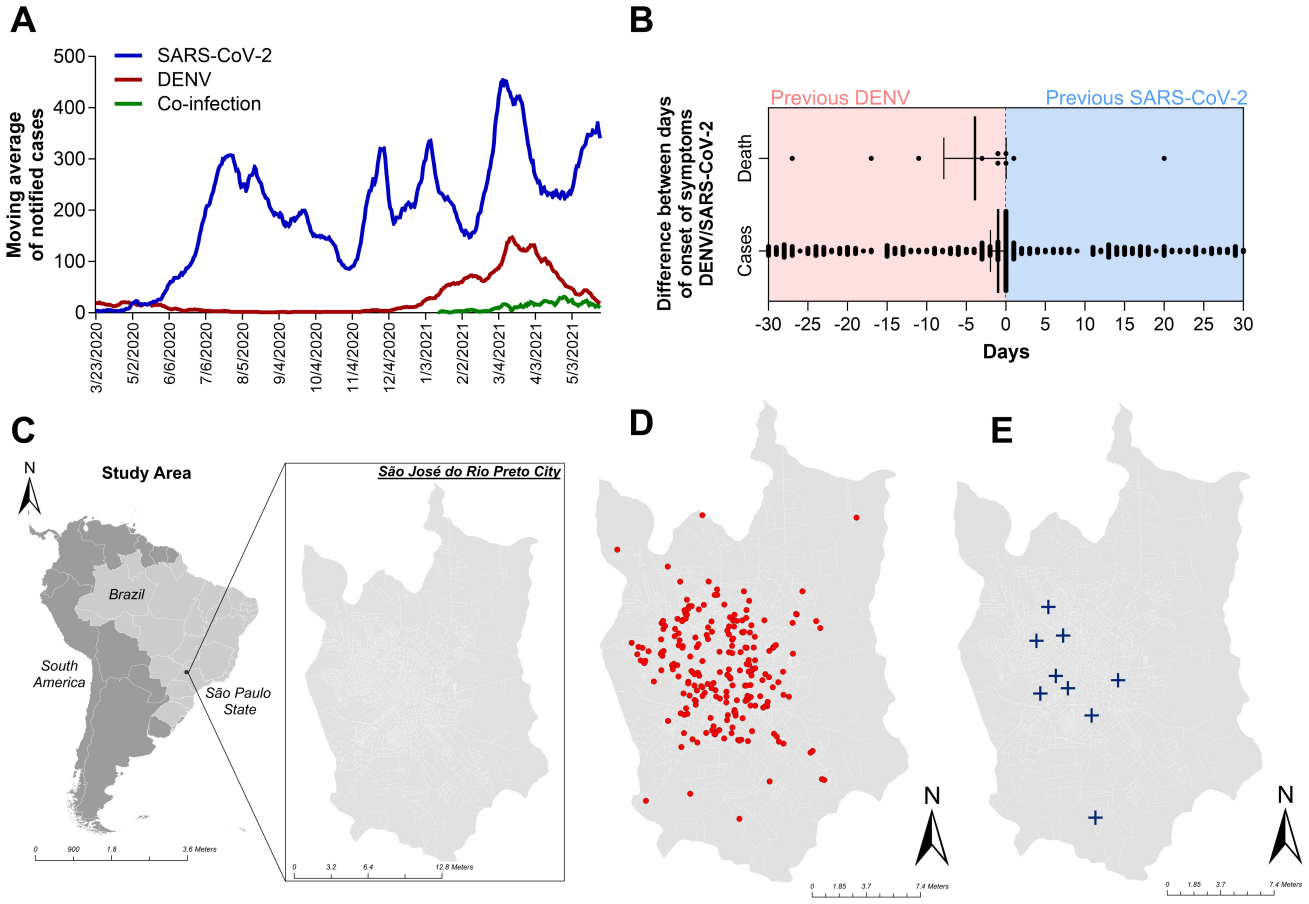
Occurrence of dengue, COVID-19, overlapping occurrence, and coinfection with both diseases in Sao Jose do Rio Preto, Sao Paulo, Brazil, from January to July 2021, according to the surveillance system databases. **(A)** Moving average of notified patients with dengue (red line) and COVID-19 (blue line) and patients with laboratory confirmation for both diseases (green line). **(B)** Differences in the onsets of symptoms reported in both databases (DENV and COVID-19) and the date of laboratory confirmation of disease. Patients whose first laboratory confirmation was that of dengue are on the red side of the graph. The blue part of the graph represents patients whose first confirmation was that of COVID-19. Only patients with a 30-day difference in laboratory confirmation of overlapping occurrence of both diseases were included in the analysis. **(C)** Geopolitical map of Brazil and São Paulo state highlighted in gray map of São Paulo state (in gray) indicating the municipality of São José do Rio Preto (SJdRP), located in the Northwest region. **(D)** Distribution of overlapping infection cases in SJdRP from January to July 2021. **(E)** Distribution of coinfection-related patient deaths in SJdRP from January to July 2021. All shapefiles used are available at https://portaldemapas.ibge.gov.br.

**Figure 2:**
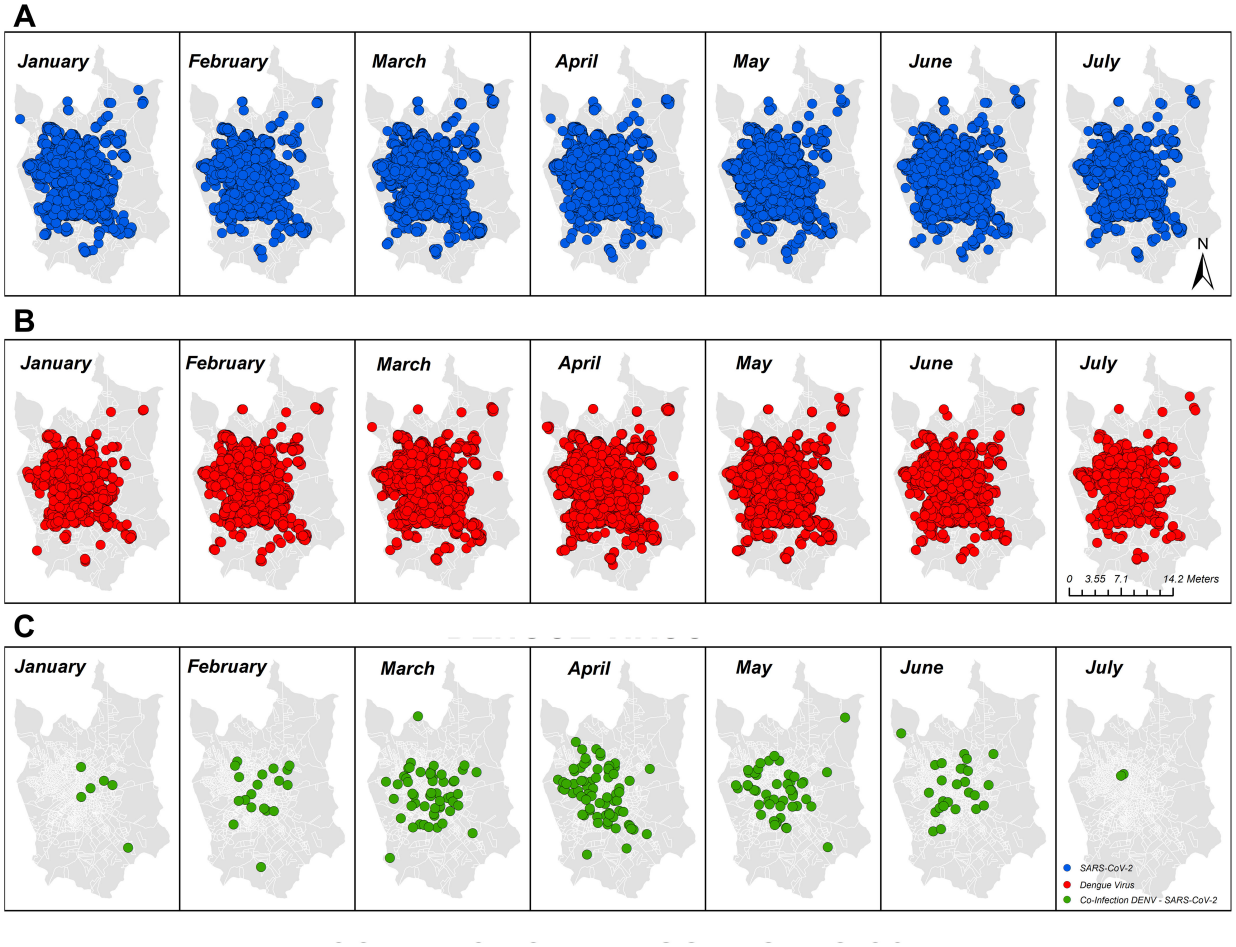
Geographic and temporal distribution of cases of COVID-19, dengue, and overlapping infection with both diseases identified in the study area of São José do Rio Preto, São Paulo, Brazil, from January to July 2021 by molecular and serologic surveillance. **(A)** COVID-19-positive patients in SJdRP; **(B)** dengue-positive patients in SJdRP; **(C)** COVID-19/dengue infection-positive patients in SJdRP. All shapefiles used are available at https://portaldemapas.ibge.gov.br.

By cross-referencing the municipality epidemiological surveillance data of COVID-19 with the dengue surveillance system in the city of Sao Jose do Rio Preto (SJdRP), we observed a simultaneous spike in cases of DENV and SARS-CoV-2 during the classic endemic period of dengue incidence in the region (**Figure 1A**), usually around April and May of each year. During the period covered by this study, we investigated 814 cases that were geographically and temporally identified in the surveillance system as coinfections. Of those, 88.5% (720) of the COVID-19-positive patients were diagnosed by RT−PCR. The other 11.5% (94), diagnosed by rapid tests (RDT), were discarded from the analysis due to a lack of information on the diagnostic assay brand/type and the impossibility of analyzing test accuracy and diagnostic performance. Among the 720 patients, 48.9% (352) were diagnosed via DENV NS1 assays, and 4 (0.5%) were also diagnosed via RT−PCR and serotyped to DENV-1 (75%) and DENV-2 (25%). When comparing both databases, patients tended to be included as DENV notifiable cases before COVID-19, because the declared onset of dengue symptoms occurred prior to the onset of symptoms reported in the COVID-19 database (**Figure 1B**). Only patients who were 30 days from the onset of symptoms were included in this analysis for overlapping infections. However, most cases were notified and tested on the same day (day 0 in the graph), indicating coinfection. The same tendency was observed in the fatal cases with overlapping infections reported. The disease distribution follows the population density in the city, with most cases (**Figure 1D**) and deaths (**Figure 1E**) occurring in the central and most populous areas of SJdRP. The temporal distributions of the total number of cases of COVID-19, total number of cases of DENV, and probable coinfection are presented in **Figure 2**.

### 3.2 DENV infection evaluation in patients with acute COVID-19 in a hospital cohort

**Figure 3:**
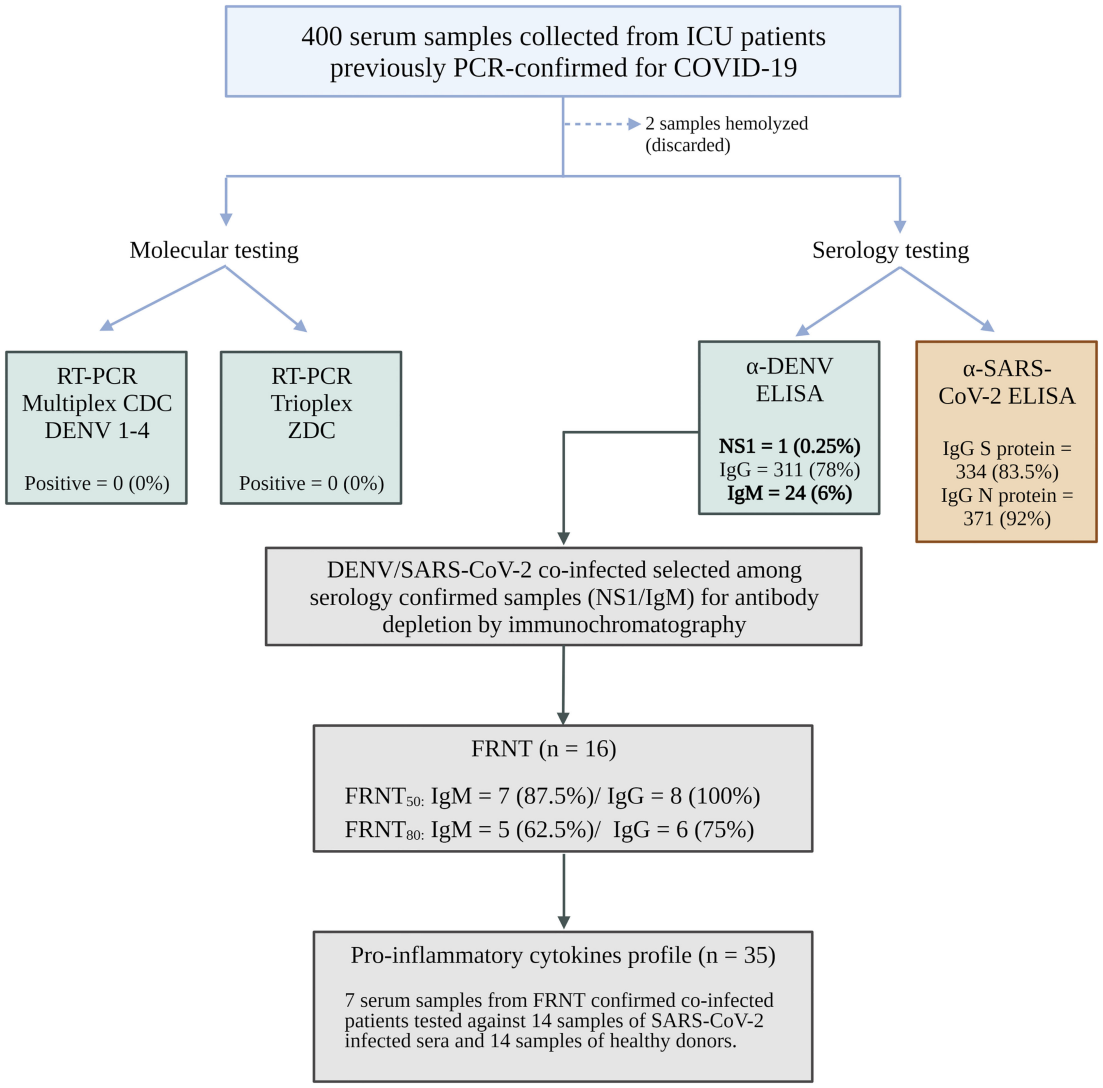
Flowchart of hospital cohort samples. Image created with BioRender.com.

To evaluate the clinical impact of possible sequential infection with DENV and SARS-CoV-2, we actively surveyed patients admitted to the Hospital de Base (HB) in SJdRP with PCR-confirmed COVID-19 who required any degree of medical assistance (ambulatory to the ICU). Per the hospital standard procedure, blood samples were collected after diagnostic confirmation to perform complementary laboratory analysis. These blood samples were also used for arboviral investigation and were tested for Zika virus and all four serotypes of DENV by RT−PCR. Of these, 6% were collected between days 0 and 7 after the onset of symptoms, 25% were collected between days 8 and 14, and the majority (69.3%) were collected from patients more than 15 days after the onset of symptoms. As observed in the epidemiological data analyzed previously, more than 94% of the samples were collected after the DENV viremic phase, which explains why none of the samples were DENV-positive according to the molecular assay. Only the data of patients who were NS1 positive were collected in the first week after the onset of symptoms (**Figure 3**).

Of all 400 individuals, 24 tested positive for dengue (dengue IgM-positive), and 366 tested negative (dengue-negative). We observed non-significant increase in overlapping incidence in white males older than 60 (95.7% and 65.2%, respectively). A lack of basic education was statistically relevant among the dengue-positive patients, with 52.4% presenting this characteristic (p=0.041). The most common comorbidity among individuals in the study was cardiopathy (60.6%), followed by obesity (43.0%) and diabetes (34.2%). Nonetheless, diabetes had a significantly lower percentage in dengue-positive patients than in dengue-negative patients (14.3% vs. 35.7%, p= 0.046) (**Table 1**).

**Table 1:** Demographic information.

Characteristics	Total no. of patients (n = 400)		Dengue-positive (n = 24)		Dengue-negative (n = 366)		p-value
	Patients	N (%)	Patients	N (%)	Patients	N (%)	
Distribution (years)							0.193
21–40	378	52 (13.76)	24	1 (4.17)	354	51 (14.41)	
41–60	378	191 (50.53)	24	11 (45.83)	354	180 (50.85)	
> 60	378	135 (35.71)	24	12 (50)	354	123 (34.75)	
Age, mean ± SD	353	54.63 ± 12.02	22	59.14 ± 13.06	331	54.33 ± 12.98	0.105
Sex							0.421
Female	376	161 (42.8)	23	8 (34.8)	353	153 (43.3)	
Male	376	215 (57.2)	23	15 (65.2)	353	200 (56.7)	
Race							0.480
White	368	327 (88.9)	23	22 (95.7)	345	305 (88.4)	
Black	368	23 (6.3)	23	1 (4.3)	345	22 (6.4)	
Brown	368	18 (4.9)	23	0 (0)	345	18 (5.2)	
Education level							
No school	287	29 (10.1)	21	0 (0)	266	29 (10.9)	0.041
Incomplete basic school	287	77 (26.8)	21	11 (52.4)	266	66 (24.8)	
Complete basic school	287	57 (19.9)	21	5 (23.8)	266	52 (19.5)	
High school	287	98 (34.1)	21	4 (19)	266	94 (35.3)	
Graduated	287	26 (9.1)	21	1 (4.8)	266	25 (9.4)	
Comorbidities							
Asthma	307	12 (3.9)	21	1 (4.8)	286	11 (3.8)	0.580
Cardiopathy	307	186 (60.6)	21	16 (76.2)	286	170 (59.4)	0.129
Diabetes	307	105 (34.2)	21	3 (14.3)	286	102 (35.7)	0.046
Down syndrome	309	1 (0.3)	21	0 (0)	288	1 (0.3)	1.000
Hematological diseases	307	2 (0.7)	21	0 (0)	286	2 (0.7)	1.000
Liver disease	307	4 (1.3)	21	0 (0)	286	4 (1.4)	1.000
Chronic kidney disease	307	24 (7.8)	21	0 (0)	286	24 (8.4)	0.389
Obesity	309	133 (43)	21	6 (28.6)	288	127 (44.1)	0.165
Pneumopathology	307	18 (5.9)	21	0 (0)	286	18 (6.3)	0.622
Puerperal period	309	4 (1.3)	21	0 (0)	288	4 (1.4)	1.000
Neurological disorders	307	16 (5.2)	21	3 (14.3)	286	13 (4.5)	0.087
Immunodepression condition	307	15 (4.9)	21	2 (9.5)	286	13 (4.5)	0.274

Baseline characteristics of patients with overlapping infections (dengue and COVID-19-positives) and COVID-19 only patients (dengue-negative).

The values are expressed as the means ± standard deviations (SDs).

p values > 0.05 indicate good model fit.

Both dengue-positive and COVID-19-only patients (dengue-negative) demonstrated a high rate of global hospitalization (95.65% and 94.9%, respectively) and a mortality rate of 50%. However, the use of invasive mechanical ventilatory support was significantly increased in dengue-positive patients, reaching 86.96%. In this group, cough was the most common initial symptom, present in 86.96% of patients (p=0.019), and 70.83% of patients experienced symptoms for more than 14 days (**Table 2**).

**Table 2:** Clinical and hospital characteristics of patients with overlapping infections (dengue and COVID-19-positives) and COVID-19 only (dengue-negative).

Characteristics	Total no. of patients (n = 400)		Dengue-positive (n = 24)		Dengue-negative (n = 366)		p-value
	Patients	N (%)	Patients	N (%)	Patients	N (%)	
Days of symptom onset							0.783
0–7	384	22 (5.73)	24	2 (8.33)	360	20 (5.56)	
8–14	384	96 (25)	24	5 (20.83)	360	91 (25.28)	
> 14	384	266 (69.27)	24	17 (70.83)	360	249 (69.17)	
Time between symptom onset and blood collection, mean [median]	353	20.37 [9.69]	22	19.50 [6.50]	331	19.35 [9.87]	0.585
Initial symptoms								
Fever	374	201 (53.74)	23	12 (52.17)	351	189 (53.85)	0.876
Cough	374	240 (64.17)	23	20 (86.96)	351	220 (62.68)	0.019
Sore throat	374	51 (13.64)	23	1 (4.35)	351	50 (14.25)	0.341
Dyspnea	374	348 (93.05)	23	21 (91.3)	351	327 (93.16)	0.668
Respiratory distress	374	278 (74.33)	23	14 (60.87)	351	264 (75.21)	0.127
Low saturation	374	363 (97.06)	23	23 (100)	351	340 (96.87)	1.000
Diarrhea	374	27 (7.22)	23	2 (8.7)	351	25 (7.12)	0.677
Abdominal pain	374	2 (0.53)	23	0 (0)	351	2 (0.57)	1.000
Vomit	374	19 (5.08)	23	2 (8.7)	351	17 (4.84)	0.329
Fatigue	374	180 (48.13)	23	11 (47.83)	351	169 (48.15)	0.976
Altered taste	374	9 (2.41)	23	0 (0)	351	9 (2.56)	1.000
Anosmia	374	16 (4.28)	23	0 (0)	351	16 (4.56)	0.612
Severity								
Hospitalization in ICU	376	357 (94.95)	23	22 (95.65)	353	335 (94.9)	1.000
Mechanical ventilation							0.124
Yes, invasive	375	297 (79.2)	23	20 (86.96)	352	277 (78.69)	
Yes, noninvasive	375	74 (19.73)	23	2 (8.7)	352	72 (20.45)	
No	375	4 (1.07)	23	1 (4.35)	352	3 (0.85)	
Hospitalization Outcome							0.936
Discharge	362	178 (49.17)	22	11 (50)	340	167 (49.12)	
Death	362	184 (50.83)	22	11 (50)	340	173 (50.88)	
Hospital admitted, mean [median]	353	6.53 [10.67]	22	2.91 [4.82]	331	6.77 [10.91]	0.313
ICU admitted, mean [median]	353	19.82 [13.19]	22	15.95 [7.59]	331	20.08 [13.43]	0.248
OD, mean [median]								
DENV IgG	353	2.38 [1.26]	22	3.06 [1.04]	331	2.33 [1.26]	< 0.001
DENV IgM	353	0.43 [0.69]	22	2.77 [1.29]	331	0.27 [0.12]	< 0.001
IgM/IgG	353	0.52 [1.26]	22	1.92 [3.92]	331	0.42 [0.76]	< 0.001

OD, optical density ratio.

Values are expressed as medians.

p values > 0.05 indicate good model fit.

Serology was performed on 398 out of 400 samples to investigate the presence of antibodies against dengue infection, and all 400 samples were tested for antibodies against the N and S proteins of SARS-CoV-2. Among the 398 samples, 311 (78%) were positive for DENV IgG, 24 (6%) were positive for DENV IgM, and 1 (0.25%) was positive for NS1. IgG antibodies against the SARS-CoV-2 N protein were detected in more than 92% (371) of the samples, while IgG antibodies against the S protein were detected in 83.5% (334) of the samples. Only two samples had insufficient volume for all the tests to be performed (**Supplementary Figure 1**). The antibodies from the 24 DENV IgM-positive samples were isolated using chromatography on a protein G column. Both DENV IgM and IgG antibodies were separated, and the success of the purification process was confirmed through another round of ELISA to ensure the efficacy of the experiment (**Supplementary Figure 2**).

A subset of 8 samples from severe patients (ICU) were subjected to purification and concentration of IgM and IgG antibodies, which were subsequently screened using the FRNT_50_ and FRNT_80_ tests to assess their neutralizing capacity against DENV 1-4. The results showed that all eight samples (100%) had neutralizing antibodies when tested with DENV IgG FRNT_50_, with 8 (100%) neutralizing for DENV-1, 7 (87.5%) for DENV-2, 2 (25%) for DENV-3, and 5 (62.5%) for DENV-4. In contrast, DENV IgM FRNT_50_ was positive in 7 of the 8 samples (87.5%), with 6 (75%) neutralizing for DENV-1, 3 (37.5%) for DENV-2, and none for DENV-3 or DENV-4. Only one sample (12.5%) did not show any neutralizing antibodies for DENV in this test. In FRNT_80_, 7 of the 8 samples (87.5%) had neutralizing antibodies when tested with DENV IgG; 5 (62.5%) neutralizing for DENV-1, 3 (37.5%) for DENV-2, and none for DENV-3 or DENV-4. Moreover, 5 of the 8 DENV IgM FRNT_80_ samples (62.5%) were neutralizing for DENV-1, 4 (50%) for DENV-1, 1 (12.5%) for DENV-2, and none for DENV-3 or 4. Some samples were positive for neutralizing antibodies for more than one serotype (**Supplementary Table 1**).

### 3.3 Cytokine expression profile differences in patients with overlapping infections

**Figure 4:**
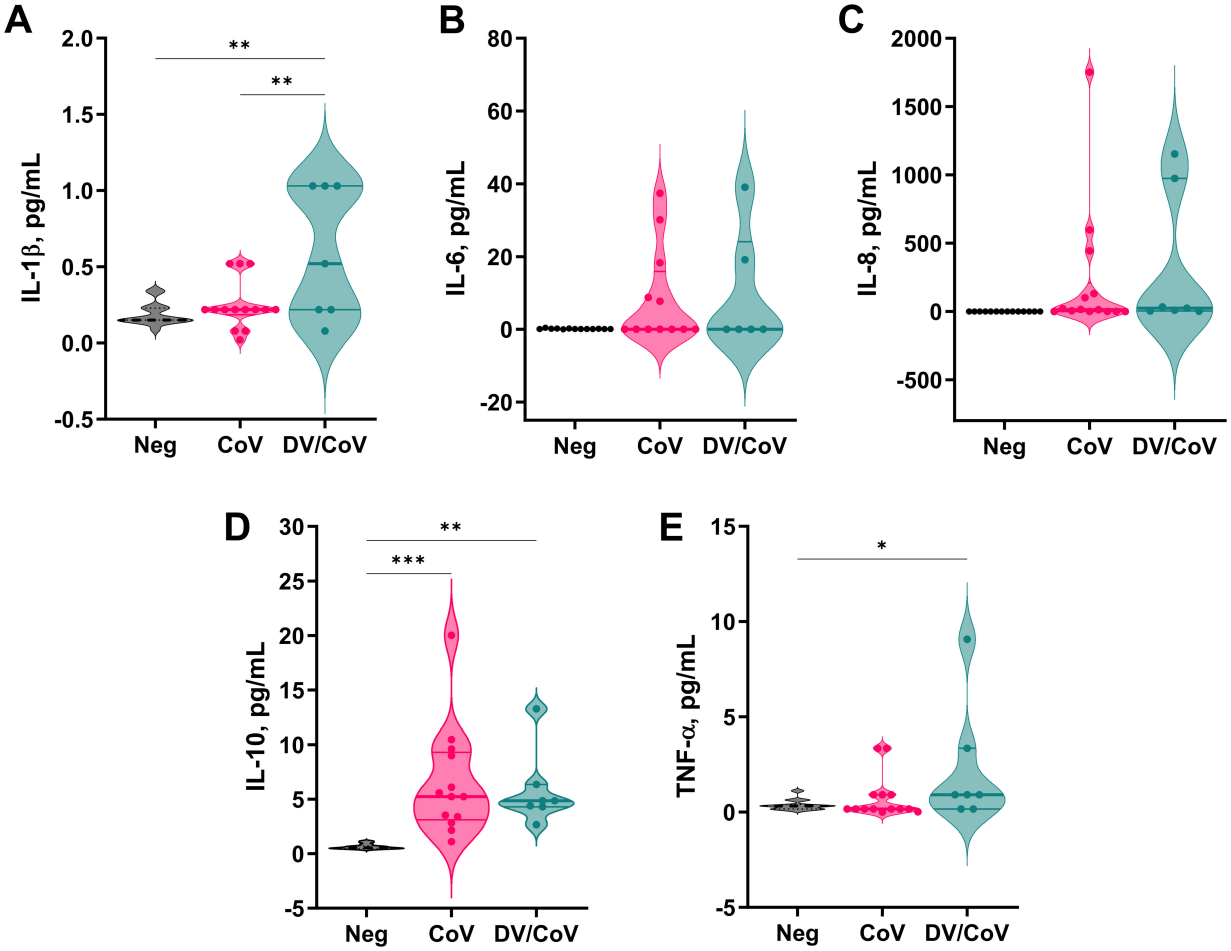
Proinflammatory cytokine profiles of a group of DENV and SARS-CoV-2 infected samples compared with samples positive only for COVID-19. The assay measured the concentrations of **(A)** IL-1β, **(B)** IL-6, **(C)** IL-8, **(D)** IL-10, and **(E)** TNF-α in the samples. *p value < 0.05; **p value < 0.01; ***p value < 0.001.

To assess the patterns of proinflammatory cytokine secretion during infection, a subset of 8 DENV/SARS-CoV-2 samples, 14 SARS-CoV-2-positive samples, and 14 healthy donor samples were analyzed. We observed an overall tendency toward cytokine secretion exacerbation for both tested groups compared to the control group. The levels of IL-1β, IL-10, and TNF-α were significantly greater. However, DENV- and SARS-CoV-2 overlapping infected patients exhibited significantly increased secretion of only IL-1β (**Figure 4**).

### 3.4 Relationship between primary or postprimary dengue and impact of acute COVID-19

When evaluating the clinical presentation of patients with primary and post-primary dengue among the DENV/CoV patients, a greater proportion of those with primary dengue had fever and cough than patients with post-primary dengue (87.50% vs. 33.33%, p=0.027 for fever). Regarding hospitalization outcomes, most patients in both groups were admitted to the ICU and required mechanical ventilation (invasive or noninvasive). Although there was no significant difference in the outcomes between the two groups, patients with post-primary dengue had longer hospital and ICU stays (4 and 20.29 days, respectively) and a higher mortality rate (60%) than patients with primary dengue, the majority of which were discharged (62.50%) (**Table 3**).

**Table 3:** Clinical and hospital characteristics of primary and postprimary dengue patients with DENV/CoV subsequent infection.

Characteristics	Dengue primary (n = 9)		Dengue postprimary (n = 15)		p-value
	Patients	N (%)	Patients	N (%)	
Sex					1.000
Female	8	3 (37.5)	15	5 (33.33)	
Male	8	5 (62.5)	15	10 (66.67)	
Age, mean [median]	7	49.29 [15.50]	15	63.73 [9.05]	0.056
Age group (years)					0.251
21–40	9	1 (11.11)	15	0 (0)	
41–60	9	5 (55.56)	15	6 (40)	
> 60	9	3 (33.33)	15	9 (60)	
Race					1.000
White	8	8 (100)	15	14 (93.33)	
Black	8	0 (0)	15	1 (6.67)	
Education level					0.078
No school					
Incomplete basic school	8	4 (50)	13	7 (53.85)	
Complete basic school	8	4 (50)	13	1 (7.69)	
High school	8	0 (0)	13	4 (30.77)	
Graduated	8	0 (0)	13	1 (7.69)	
Days of symptom onset					0.643
0–7	9	1 (11.11)	15	1 (6.67)	
8–14	9	1 (11.11)	15	4 (26.67)	
> 14	9	7 (77.78)	15	10 (66.67)	
Days of symptoms, mean [median]	22	19.50 [6.50]	331	20.42 [9.87]	0.585
Initial symptoms					
Fever	8	7 (87.5)	15	5 (33.33)	0.027
Cough	8	7 (87.5)	15	13 (86.67)	1.000
Sore throat	8	0 (0)	15	1 (6.67)	1.000
Dyspnea	8	8 (100)	15	13 (86.67)	0.526
Respiratory distress	8	4 (50)	15	10 (66.67)	0.657
Low saturation	8	8 (100)	15	15 (100)	NA
Diarrhea	8	0 (0)	15	2 (13.33)	0.526
Abdominal pain	8	0 (0)	15	0 (0)	NA
Vomit	8	0 (0)	15	2 (13.33)	0.526
Fatigue	8	4 (50)	15	7 (46.67)	1.000
Altered taste	8	0 (0)	15	0 (0)	NA
Anosmia	8	0 (0)	15	0 (0)	NA
Severity					
Hospitalization in ICU	8	7 (87.5)	15	15 (100)	0.348
Mechanical ventilation					
Yes, invasive	8	7 (87.5)	15	13 (86.67)	0.230
Yes, noninvasive	8	0 (0)	15	2 (13.33)	
No	8	1 (12.5)	15	0 (0)	
Hospitalization outcome					0.361
Discharge	8	5 (62.5)	15	6 (40)	
Death	8	2 (25)	15	9 (60)	
Hospital admitted, mean [median]	7	2.40 [3.54]	15	4.00 [7.07]	0.591
ICU admitted, mean [median]	7	13.93 [5.81]	15	20.29 [9.53]	0.190

NA, not available.

Values are expressed as medians.

p values > 0.05 indicate a good model fit.

Finally, among the patients who were negative for acute dengue, we analyzed the impact of dengue immunity in light of a new SARS-CoV-2 infection. We observed that those with previous dengue infection had a median age of 55.76 years (p=0.039) and a high school education (35.41%). Although the most common COVID-19 symptoms, such as fever, cough, and low oxygen saturation, were similar for both groups, individuals with previous dengue infection had a greater percentage of dyspnea (94.81% vs. 88.31%, p=0.043) and a lower percentage of diarrhea (5.19% vs. 14.29%, p=0.006) and vomiting (6.30% vs. 0.00%, p=0.017) compared to those who were dengue-naïve. In terms of hospitalization, there was no significant difference between the two groups regarding ICU admission or length of hospital stay. Although non-significant, there was a trend toward a greater percentage of invasive mechanical ventilation use among those naïve to dengue infection (81.58% vs. 77.57%). Additionally, the two groups had no significant differences in hospitalization outcomes (discharge vs. death) (**Table 4**).

**Table 4:** Clinical and hospital characteristics of dengue-naïve patients and patients with previous dengue among the COVID-19-only group.

Characteristics	Dengue-naïve (n = 83)		Previous dengue (n = 283)		p-value
	Patients	N (%)	Patients	N (%)	
Sex					0.500
Female	77	26 (33.77)	272	126 (46.32)	
Male	77	51 (66.23)	272	146 (53.68)	
Age, mean [median]	77	51.74 [14.98]	272	55.76 [12.32]	0.039
Age group (years)					0.006
21–40	78	20 (25.64)	272	31 (11.4)	
41–60	78	32 (41.03)	272	144 (52.94)	
> 60	78	26 (33.33)	272	97 (35.66)	
Race					0.554
White	72	63 (87.5)	269	240 (89.22)	
Black	72	6 (8.33)	269	14 (5.2)	
Brown	72	3 (4.17)	269	15 (5.58)	
Education level					0.994
No school	72	6 (8.33)	209	23 (11)	
Incomplete basic school	72	13 (18.06)	209	51 (24.4)	
Complete basic school	72	9 (12.5)	209	41 (19.62)	
High school	72	20 (27.78)	209	74 (35.41)	
Graduated	72	5 (6.94)	209	20 (9.57)	
Initial symptoms					
Fever	77	43 (55.84)	270	145 (53.7)	0.739
Cough	77	44 (57.14)	270	175 (64.81)	0.218
Sore throat	77	9 (11.69)	270	41 (15.19)	0.441
Dyspnea	77	68 (88.31)	270	256 (94.81)	0.043
Respiratory distress	77	55 (71.43)	270	206 (76.3)	0.383
Low saturation	77	75 (97.4)	270	261 (96.67)	1.000
Diarrhea	77	11 (14.29)	270	14 (5.19)	0.006
Abdominal pain	77	0 (0)	270	2 (0.74)	1.000
Vomit	77	0 (0)	270	17 (6.3)	0.017
Fatigue	77	40 (51.95)	270	129 (47.78)	0.518
Altered taste	77	1 (1.3)	270	8 (2.96)	0.418
Anosmia	77	2 (2.6)	270	14 (5.19)	0.340
Severity					
Hospitalization in ICU	77	74 (96.1)	272	257 (94.49)	0.773
Mechanical ventilation					0.092
Yes, invasive	76	62 (81.58)	272	211 (77.57)	
Yes, non invasive	76	12 (15.79)	272	60 (22.06)	
No	76	2 (2.63)	272	1 (0.37)	
Hospitalization outcome					0.577
Discharge	74	34 (45.95)	262	130 (49.62)	
Death	74	40 (54.05)	262	132 (50.38)	
Hospital admitted, mean [median]	72	7.85 [11.92]	266	6.46 [10.78]	0.716
ICU admitted, mean [median]	75	18.35 [10.84]	277	19.55 [14.30]	0.616

Values are expressed as medians.

p values < 0.05 indicate good model fit.

## 4 Discussion

As of March 2024, Brazil was the sixth-leading country in terms of confirmed cases and the second-leading country in the world in terms of absolute deaths from COVID-19 (World Health Organization, 2020). The impact on the national health system was abysmal and affected well-established non-COVID-19 public healthcare attention programs and leading to the complete shutdown of services and underreporting of other important infectious diseases (Rabiu et al., 2021). Dengue has been a health concern since its introduction in Brazil, but with the COVID-19 pandemic, a decrease in the number of registered cases and deaths of dengue was observed in 2021 (Lu et al., 2021; Souza and Romano, 2022). According to data from the Brazilian Ministry of Health, there were more than 500,000 probable cases of dengue in Brazil in 2021, with 230 confirmed deaths. Compared to 2020, there was a reduction of 51.9% and 43.6% in the number of registered cases and deaths, respectively (Souza and Romano, 2022). However, in the southeastern region of Brazil, where the city of São José do Rio Preto (SJdRP) is located, the incidence rate of dengue was still high, with 197.2 cases per 100,000 inhabitants by week 25 of 2021 (Ministry of Health of Brazil and Secretaria de Vigilância em Saúde, 2021). Importantly, SJdRP is a hotspot for arboviruses, as one of the Brazilian centers with the best-implemented surveillance systems for dengue and a well-documented historical progression of the disease (Rocco et al., 2012; Pinheiro et al., 2018; Chiaravalloti-Neto et al., 2019). This can explain the disparities in numbers from the region compared to the whole country, which makes SJdRP a valuable resource because of the interactions of both viruses that have been elicited in the scientific community since the beginning of the pandemic.

During the COVID-19 pandemic, SJdRP presented many reports of undifferentiated acute febrile illness, noting both diseases concomitantly and reporting a coincident increase in the number of reported cases for both DENV and SARS-CoV-2 during the endemic period for dengue in the region (**Figure 1A**). After viral diagnosis by RT−qPCR and/or DENV NS1 detection, patients were found to be temporally distributed. Our study revealed a death rate of 5.6% (14/285) related to SARS-CoV-2 and DENV overlapping infection; however, this rate is variable in the literature and has been described elsewhere as higher than 16%, which is significantly greater than the global death rate of a single infection of either disease (Prapty et al., 2023). Similar findings of DENV/SARS-CoV-2 coinfection have been reported in Colombia, the Philippines, China, Thailand, Mexico, and different Brazilian states (Ratnarathon et al., 2020; Saipen et al., 2021; Schulte et al., 2021; Acosta-Pérez et al., 2022; Del Carpio-Orantes et al., 2022; Prapty et al., 2022; Tangsathapornpong and Thisyakorn, 2023). The first recorded case of a human coinfection of SARS-CoV-2 and DENV occurred in Brazil and occurred in early 2020, when RT−qPCR was used to detect both viruses concurrently. Despite being diagnosed with both diseases, the patient did not develop severe symptoms associated with either infection (Bicudo et al., 2020). The simultaneous detection of spatial clusters of dengue and COVID-19 transmission was also reported by Pereira and collaborators (Pereira et al., 2021) in a study performed in Presidente Prudente, a municipality 273 km away from São José do Rio Preto in the state of São Paulo.

As the symptomatology and laboratory findings of dengue and COVID-19 can overlap and complicate accurate diagnosis, risk assessment, and treatment (Dutta et al., 2023), we investigated a hospital cohort of COVID-19 patients to actively detect dengue occurrence. Diabetes, a preexisting metabolic condition known to exacerbate many infectious and inflammatory diseases, is one of the most common comorbidities found in coinfected patients and poses a significant risk factor for severe disease and higher case fatality rates, as observed in previous studies (Tsheten et al., 2021; León-Figueroa et al., 2022). Recent studies have also suggested that diabetes increases the transmissibility of other arboviruses, which may impact the population infection rate (Azar et al., 2022). In our study, diabetes was a significant comorbidity (p=0.046) in the overlapping DENV/SARS-CoV-2 infected group (**Table 1**). Clinically, cough was the initial symptom with greater significance among this group, presenting in 86.96% of patients (p=0.019). Some studies have reported that 25% of patients with confirmed dengue present with cough, and 20% have upper respiratory tract symptoms. Similarly, COVID-19 may manifest as fever with muscle and joint pain without respiratory symptoms, especially in infants (Rabiu et al., 2021). Due to overlapping symptoms, most patients must be tested for both diseases. Additionally, in this study, 70.8% of the patients experienced symptoms for more than 14 days, with a median hospitalization time of 4.8 days and an average ICU stay of 7.6 days. The duration of hospital stay observed in our study was shorter than that reported in other studies, which reported a mean hospital stay of 11.4 days for patients with overlapping infections (El-Qushayri et al., 2022). Both the dengue-positive and dengue-negative groups demonstrated a high rate of global hospitalization (95.65 and 94.9%, respectively) and a mortality rate of 50%. However, 86.96% of DENV/CoV patients required invasive mechanical ventilatory support, indicating a potential impact on patient disease progression. Previous studies have reported a mortality rate of up to 80% in ICU-coinfected patients treated with mechanical ventilation, but in this study, it did not increase the number of deaths (MaChado and Kimura, 2022) (**Table 2**).

The sera were obtained 19.5 days after the onset of symptoms, on average. Arboviral RT−qPCR tests for the four dengue serotypes (DENV1−4), Zika virus (ZIKV), and Chikungunya virus (CHIKV)) were performed because of their similar early-stage symptoms, including fever, myalgia, and headaches (Mota et al., 2021). However, all test results were negative—a phenomenon that can be explained by the timing of sample collection, as viral loads are typically detectable within the first five days of symptom onset (Guzman and Harris, 2015). We detected DENV IgM-positive results by ELISA in 24 (6%) patients and DENV NS1-positive results in 1 (0.25%) patient (**Figure 3**). Among all the serum samples, 311 (78%) were DENV IgG-positive according to ELISA, which corresponded to the disease prevalence in the region (Chiaravalloti-Neto et al., 2019) and classified the majority of our study population as having preexisting exposure to DENV. Nevertheless, cross-reactivity in antibody tests was reported between SARS-CoV-2 and dengue virus early in the pandemic, and dengue-like syndromes with thrombocytopenia and false IgM-positivity have been described (Nacher et al., 2020; Dutta et al., 2023).

To exclude the possibility of false-positive dengue serology in this study, samples from the overlapping infected group were subjected to depletion and purification of IgM and IgG antibodies. A subset of 8 samples were tested using FRNT, which is considered the gold standard for measuring neutralizing antibody responses against DENV infection (World Health Organization, 2007). Among these samples, 7 (87.5%) were positive for IgM in FRNT_50,_ and all 8 (100%) were positive for IgG. Only one sample was negative for DENV-neutralizing antibodies in the FRNT_50_. However, serological cross-reactivity between DENV and SARS-CoV-2 has been largely discussed in the literature in the past three years, with mixed conclusions (Ioannidis and Contopoulos-Ioannidis, 2023). Several works have reported the inefficacy of lateral-flow assays (LFAs) in identifying properly infectious agents using both DENV and COVID-19 assays (Lustig et al., 2021; Santoso et al., 2021; Dutta et al., 2023). Conversely, studies using dengue samples from endemic regions such as Puerto Rico (Munoz-Jordan et al., 2022) and Colombia (Faccini-Martínez Á et al., 2020) or from prepandemic sera banks (Spinicci et al., 2020) have shown minimal cross-reactivity with SARS-CoV-2 when tested with ELISAs. However, misdiagnosis via the serologic DENV assay is not exclusive to possible cases of COVID-19 infection. The combination of NS1 antigen detection with the IgM/IgG antibody assay has been recommended for several years to improve the accuracy of serological tests, especially rapid tests (Blacksell et al., 2011; Lee et al., 2019). Interestingly, laboratory features – such as neutrophil, platelet counts, neutrophil-lymphocyte ratio (NLR), and neutrophil-lymphocyte*platelet ratio (NLPR), can been used to differentiate COVID-19 from DENV infections without serology (Osuna-Ramos et al., 2022).

High levels of inflammatory cytokines, a pathological state termed cytokine release syndrome (CRS), can result in multiorgan failure and increased mortality in COVID-19 patients and may contribute to the severity of dengue (Dayarathna et al., 2020; Irwinda et al., 2021). Many cytokines are associated with both dengue and COVID-19 clinical disease aggravation. A dramatic increase in IL-6 level has been associated with COVID-19 and appears to significantly impact disease progression compared to that of SDD (Dayarathna et al., 2020; Moore and June, 2020). In addition to being related to viral infection, studies have shown that the overproduction of IL-6 is associated with lung damage, which can explain why IL-6 receptor blockers are among the most-suggested treatments for COVID-19 (Stone et al., 2020; Gordon et al., 2021; Salama et al., 2021). Our findings indicate a non-significant tendency for higher levels of IL-6 in both the DENV/COVID-19 infected and COVID-19-only groups than in the donor group (**Figure 4**). Commonly associated with IL-6 secretion, IL-8 levels showed the same tendency as those of IL-6 in COVID-19 patients. Previous studies on severe acute respiratory syndrome (SARS) revealed the same pattern of increased concentrations of IL-6 in mildly affected patients, but the concentrations were significantly similar in control subjects. Researchers also observed spiked production in severely affected patients, linking IL-6 levels to SARS severity (Zhang et al., 2004). Importantly, our overlapping infected group was selected by test positivity, not by clinical patterns, disease severity, or outcome. The lower number of patients analyzed and the nonuniformity of the clinical timeline and severity can explain the differences observed between our study and the literature and must be considered a limitation for this analysis.

Conversely, IL-10 levels were significantly greater in both infected groups than in the control group, and this difference was more pronounced in COVID-19-only infected patients with a median concentration > 5 pg/mL. IL-10 is a critical cytokine in dengue infection and its levels are reported to be strongly increased in both DF and SDD patients (Dayarathna et al., 2020). However, it is still a severity biomarker for COVID-19, with significantly greater levels in ICU patients than in non-ICU patients (Huang et al., 2020). Elevated levels of IL-10 are associated with T-cell apoptosis in acute dengue (Malavige et al., 2012) and T-cell exhaustion in COVID-19, presumably via overactivation and proliferation (Diao et al., 2020). Although several hypotheses have proposed the pathogenic role of IL-10 in both viral infections, the occurrence of subsequent infections does not exacerbate cytokine production compared to SARS-CoV-2 infection alone. Interestingly, the only cytokine whose levels significantly differed between COVID-19-infected patients and SARS-CoV-2/DENV-infected patients was interleukin 1β (IL-1β). The IL-1 family plays a central role in the regulation of immune and inflammatory responses, and infection-induced pyroptosis is a type of lytic cell death triggered by an inflammatory signal and characterized by cellular swelling, lysis, and the release of inflammatory cytokines, such as IL-1β and IL-18 (Bergsbaken et al., 2009). Excessive inflammation can lead to tissue damage, vascular leakage, and, consequently, the exacerbation of the disease (Pan et al., 2019). In this sense, the IL-1β pathway is well-known in DENV-infected macrophages, monocytes, and, more recently, dermal fibroblasts (Tan and Chu, 2013; Wu et al., 2013; Wei et al., 2023). Similarly, a study describing the *in vitro* infection of human primary monocytes indicated that SARS-CoV-2 also engages inflammasomes and promotes pyroptotic events, leading to enhanced levels of IL-1β (Ferreira et al., 2021). Such exacerbation of the inflammatory response during infection with DENV and SARS-CoV-2 can explain the increased levels of secreted IL-1β observed in our study.

Finally, TNF-α was present in lower levels, probably because the serum was collected in the postacute phase of the infection. Generally, TNF-α levels peak in the early phases of infection and decrease significantly in the second week, except in patients with SDD (Dayarathna et al., 2020). Recent studies have reported greater viral replication and IL-10, IL-6, and IL-1β levels in patients with a history of SDD than in individuals with a history of milder dengue disease (Kamaladasa et al., 2019). In a city where the DENV prevalence is almost 80%, these data could indicate that patients infected with SARS-CoV-2 have the worst prognosis. However, although we observed a significant increase in the use of invasive mechanical ventilatory support, it did not reflect the death rate among DENV/SARS-CoV-2-infected patients. Studies with larger sample sizes are required to further assess the impact of subsequent infections on cytokine production and its correlation with disease progression.

Interestingly, recent epidemiological evidence suggests that regions with a higher incidence of dengue, such as Brazil, and consequently, individuals with prior exposure to the virus may confer immunological protection against COVID-19 (Silvestre et al., 2021). However, we identified that all our secondary dengue patients progressed to severe illness and required ICU hospitalization with longer stays in the hospital and ICU (4 and 20.29 days, respectively), with 60% of patients progressing to death. Nonetheless, 87.5% of the primary dengue patients also required intensive care unit (ICU) hospitalization, but the discharge rate was 62.5%. Among these individuals, fever was the most common initial symptom (87.50%) (**Table 3**). Among individuals who had previous dengue infection and those who were dengue-naïve within the COVID-19-only group, there was no significant difference between the two groups regarding ICU admission or length of hospital stay, but there was a trend toward a greater percentage of invasive mechanical ventilation use among those naïve for dengue infection (81.58% vs. 77.57%) (**Table 4**).

As Hickam’s dictum very directly states, “patients can have as many diseases as they damn well please” (Borden and Linklater, 2013). This does not mean that it is simple to determine the dynamics of pathogen interactions. What are the minor or major players in the modulation of the disease outcome? The exacerbated symptoms among the primary infections could be associated with the proinflammatory states triggered by SARS-CoV-2 and DENV infections, leading to tissue damage and vascular leakage. However, the ability of SARS-CoV-2 antibodies to neutralize DENV1 and DENV2 *in vitro* can also indicate cross-protection. Conversely, our study revealed no significant difference in the risk of death for any possible scenarios (primary dengue vs. postprimary dengue or dengue-naïve vs. previous dengue infection). Future studies are necessary to identify interactions between these diseases and other arboviruses, especially in regions with preexisting immunity to dengue and other pathogens. This knowledge is vital to assist in adequate medical approaches for these types of cases and, consequently, for applying the most appropriate patient management and effective surveillance of these pathogens.

## Data Availability

The original contributions presented in the study are included in the article/**Supplementary Material**. Further inquiries can be directed to the corresponding author.
