# Supplementary material for: Dengue and SARS-CoV-2 co-circulation and overlapping infections in hospitalized patients
Source: Front Cell Infect Microbiol. 2024 Nov 8;14:1429309. doi: 10.3389/fcimb.2024.1429309 (PMC11582011; doi:10.3389/fcimb.2024.1429309)
Supplement: Supplementary file 1 [file DataSheet1.docx]

Supplementary Material


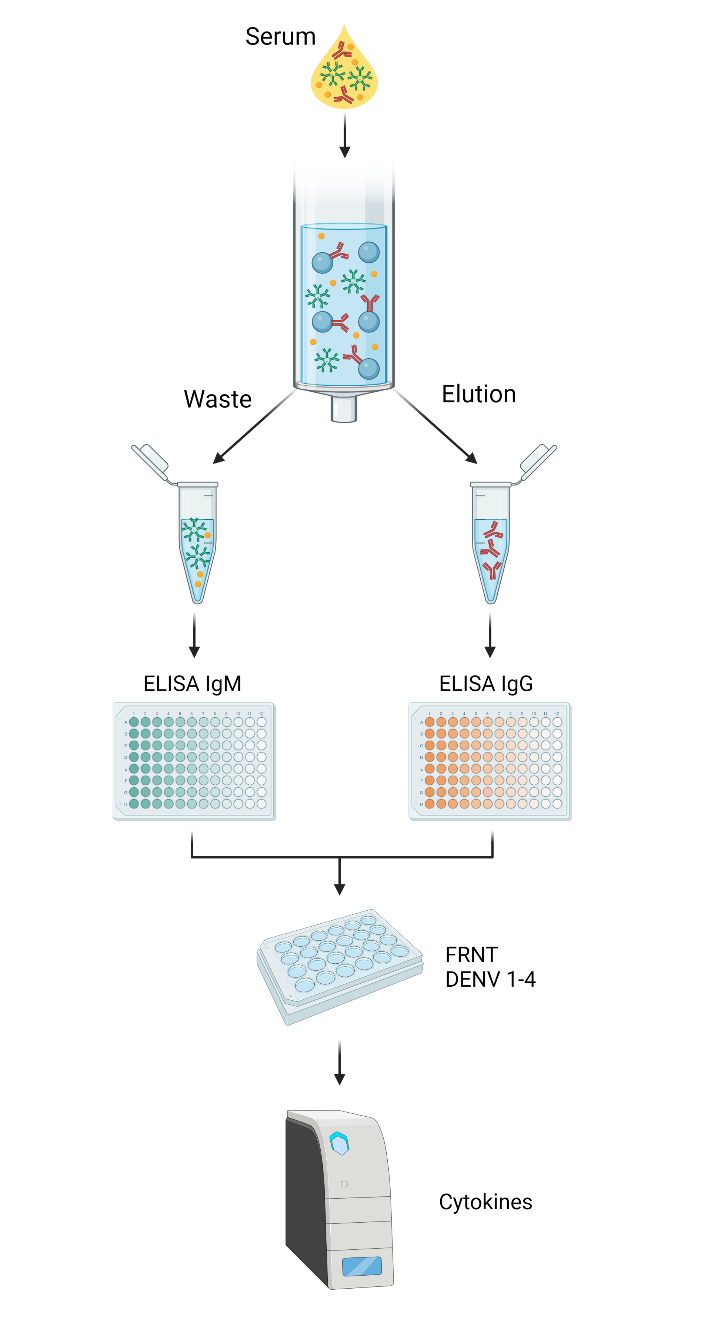


**Supplementary Figure 1.** Schematic diagram of sample preparation for antibody depletion and analysis. Image created with BioRender.com.


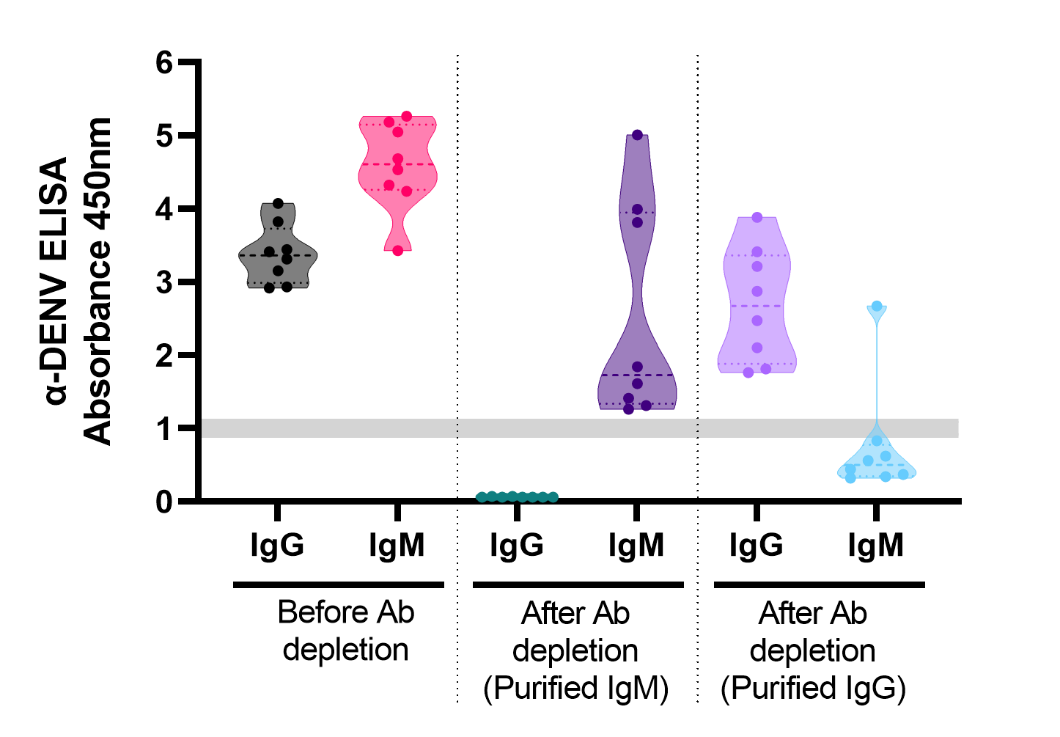


**Supplementary Figure 2.** Antibody depletion of the DENV IgM- and NS1-positive samples. Indirect IgG ELISA and IgM capture ELISAs were performed before and after antibody purification, and both waste and elution solutions were used to quantify the presence of α-DENV antibodies. The gray area indicates indeterminate values (0.9-1.1).

**Supplementary Table 1.** Focus reduction neutralization test (FRNT50 and FRNT80) results for the DENV IgM- and NS1-positive samples

| Sera (ID) | DENV-1 FRNT_50_ | DENV-1 FRNT_80_ | DENV-2 FRNT_50_ | DENV-2 FRNT_80_ | DENV-3 FRNT_50_ | DENV-3 FRNT_80_ | DENV-4 FRNT_50_ | DENV-4 FRNT_80_ |
| --- | --- | --- | --- | --- | --- | --- | --- | --- |
| After antibody depletion (Purified IgG) |  |  |  |  |  |  |  |  |
| 2804 | > 20 | 0 | > 20 | 0 | 0 | 0 | 0 | 0 |
| 2722 | > 20 | > 20 | > 20 | > 20 | > 20 | 0 | > 20 | 0 |
| 2924 | > 20 | 0 | > 20 | > 20 | > 20 | 0 | > 20 | 0 |
| 2774 | > 20 | > 20 | > 20 | 0 | 0 | 0 | 0 | 0 |
| 2787 | > 20 | > 20 | 0 | 0 | 0 | 0 | 0 | 0 |
| 2807 | > 20 | > 20 | > 20 | 0 | 0 | 0 | > 20 | 0 |
| 2878 | > 20 | 0 | > 20 | 0 | 0 | 0 | > 20 | 0 |
| 2772 | > 20 | > 20 | > 20 | > 20 | 0 | 0 | > 20 | 0 |
| After antibody depletion (Purified IgM) |  |  |  |  |  |  |  |  |
| 2804 | > 20 | > 20 | 0 | 0 | 0 | 0 | 0 | 0 |
| 2722 | > 20 | > 20 | 0 | 0 | 0 | 0 | 0 | 0 |
| 2924 | 0 | 0 | > 20 | > 20 | 0 | 0 | 0 | 0 |
| 2774 | > 20 | 0 | > 20 | 0 | 0 | 0 | 0 | 0 |
| 2787 | > 20 | > 20 | > 20 | 0 | 0 | 0 | 0 | 0 |
| 2807 | > 20 | > 20 | 0 | 0 | 0 | 0 | 0 | 0 |
| 2878 | 0 | 0 | 0 | 0 | 0 | 0 | 0 | 0 |
| 2772 | > 20 | 0 | 0 | 0 | 0 | 0 | 0 | 0 |
